# Productive Performance and Meat Characteristics of Kids Fed a Red Orange and Lemon Extract

**DOI:** 10.3390/ani11030809

**Published:** 2021-03-13

**Authors:** Angela Salzano, Sara Damiano, Livia D’Angelo, Gabriele Ballistreri, Salvatore Claps, Domenico Rufrano, Aristide Maggiolino, Gianluca Neglia, Pasquale De Palo, Roberto Ciarcia

**Affiliations:** 1 Department of Veterinary Medicine and Animal Productions, University of Naples “Federico II”, 80137 Naples, Italy; angela.salzano@unina.it (A.S.); sara.damiano@unina.it (S.D.); livia.dangelo@unina.it (L.D.); neglia@unina.it (G.N.); roberto.ciarcia@unina.it (R.C.); 2 Council for Agricultural Research and Economics (CREA)—Research Centre for Olive, Fruit and Citrus Crops, 95024 Acireale, Italy; gabriele.ballistreri@crea.gov.it; 3 Council for Agricultural Research and Economics—Research Centre for Animal Production and Aquaculture, 85051 Bella Muro, Italy; salvatore.claps@crea.gov.it (S.C.); drufrano@tiscali.it (D.R.); 4 Department of Veterinary Medicine, University “Aldo Moro” of Bari, 70010 Valenzano, Italy; pasquale.depalo@uniba.it

**Keywords:** goat meat, red orange and lemon extract, bioflavonoids, oxidative status, fatty acid profile, meat quality

## Abstract

**Simple Summary:**

Small ruminant farming can potentially adopt low-input strategies by using agro-industrial byproducts as alternative feeding sources. Byproducts are natural, and thus are preferred by consumers because most of them have antioxidant activity that may improve different aspects linked to meat quality and human health. For this reason, a red orange and lemon extract, (RLE) rich in anthocyanins, is tested as an oral additive on kids’ meat. No differences were recorded on animal performance, but on meat, RLE inclusion improves juiciness and reduces color deterioration. Moreover, RLE reduced cooking loss, and meat quality is positively influenced, due to both delaying lipid oxidation and a better fatty acid profile resulting in healthier meat for human consumption.

**Abstract:**

This study evaluates the animal performance and meat characteristics of 60 Saanen suckling kids daily fed a red orange and lemon extract (RLE), rich in anthocyanins. In our methodology, after colostrum administration, animals are randomly assigned to two treatments: Treatment group (Group RLE; *n* = 30) that received RLE (90 mg/kg live body weight) as oral food additive, and a control group (Group CON; *n* = 30) that received a standard diet. Animals are slaughtered after 40 days. The RLE administration did not influence daily weight gain, carcass measurements, or incidences (expressed as a percentage) of different anatomical regions on the whole carcass weight. On the contrary, RLE supplementation significantly improved the oxidative profile of the meat seven days after slaughtering, as demonstrated by the reduced levels of thiobarbituric acid reactive substances (TBARS; *p* < 0.01) and hydroperoxides (*p* < 0.01) in Group RLE compared to Group CON. A significant influence of RLE administration is observed on day 7 for yellowness (*p* < 0.01). There are also lower saturated and higher monounsaturated and polyunsaturated fatty acids concentration in Group RLE meat (*p* < 0.01), which also shows lower atherogenic and thrombogenic indexes (*p* < 0.01) compared to Group CON. The study demonstrates that the supplementation of a diet with RLE rich in anthocyanins is effective to improve the meat quality.

## 1. Introduction

Goats are among the earliest animals domesticated by humans [1]. This species is well adapted to harsh environments and consumes limited feed to produce high-quality products. Furthermore, it is usually reared in marginal areas, representing an important source of animal-derived protein in both developed and developing countries. Indeed, goat meat is high in protein and low in fat, and this feature makes this food an attractive alternative to red meat to consumers. Nowadays, the meat industry is facing unprecedented challenges linked to increased production costs. To overcome this problem, alternative feeding resources, such as agro-industrial byproducts, are used. The re-utilization of agri-food industry wastes as additives for livestock is well accepted by consumers, because natural additives are considered safe and healthy [2]. Moreover, recently, the food processing industries, considering their low environmental impact, are interested in using natural antioxidants rather than synthetic counterparts [3,4]. In the past years, the attention has been focused on natural feeding additives, especially from plants [3], such as olive leaves [5], cardoon meal, ramie [6], and sea buckthorn pomace [7]. One of the main problems in the meat industry is the lipid and protein oxidative deterioration that could affect flavor, color, and quality of meat, with a negative impact on meat quality and shelf life [8]. In this context, the supplementation of ruminants’ diet with antioxidants is considered a good strategy to overcome this problem trying to lowering oxidative deterioration and improve the fatty acid composition of the meat [9]. Nowadays, many studies have been carried out to develop new natural antioxidants, such as polyphenols [10] or Vit. E [11] in growing kids.

Anthocyanins are known as flavonoids and are particularly present in plants. The latter is responsible for the bright and attractive orange, red, purple, and blue colors of most fruits, vegetables, flowers, and some cereal grains. Although anthocyanins were only known for their coloring properties, in the last few years, an increasing interest has been raised around them because of their possible health benefits as dietary antioxidants [12]. At the cellular level, anthocyanins protect proteins and lipids from direct DNA damages, through their antioxidant action [13]. It was also hypothesized the capacity to activate enzymes, such as glutathione reductase, glutathione peroxidase, and glutathione S-transferase able to reduce oxidative stress [14]. Numerous in vivo studies have shown that the cyanidin 3-glucoside, most present in red oranges, could reduce body weight, fat accumulation, and high fat induced diabetes [15,16]. Therefore, flavonoid-rich foods may help to boost overall health by offering an array of nutrients. Lemon fruit is well known to have many beneficial effects on human health, due to its higher flavonoids content (mainly in flavanones) and other bioactive compounds, such as phenolic acids (hydroxycinnamic acids) and vitamins (ascorbic acid). Several studies have assessed that polyphenols possess antioxidant and anti-inflammatory properties, in particular, it has been shown the flavonoids’ ability present in a lemon extract to regulate lipid metabolism [17].

A recent study has demonstrated significant antioxidant activity of the new extract, used in the present work on the counteract the renal toxicity induced by mycotoxin [18]. In particular, it was demonstrated that in the kidney of Sprague_Dawley rats, the biochemical and oxidative stress parameters modified during the treatment with ochratoxin A, were significantly restored during the treatment with a red orange and lemon extract (RLE) rich in bioflavonoids.

Since dietary inclusion of bioactive substances characterized by antioxidant activity, can protect cell tissues from lipoperoxidative damage, prevent lipid oxidation and restore animal oxidative balance [19], we hypnotized that the addition of an RLE obtained from red orange and lemon processing wastes on suckling Saanen kids may improve animal performances, carcass characteristic, meat’s antioxidant status, colorimetric profile, texture analysis, and meat fatty acid composition.

## 2. Materials and Methods

The trial was authorized by the Animal Welfare Body of the University of Naples Federico II (PG/2019/0028161 of 03/19/2019).

### 2.1. Animal Management and Feeding

The experimental procedures were carried out in the experimental farm of the Council for Agricultural Research and Economics, Research Centre for Animal Production and Aquaculture (CREA-ZA) located in Bella (PZ, Italy), in the South of Italy, at 40°75′ latitude and 15°67′ W longitude, and 802 m above sea level. The trial was carried out on 60 single male and female kids. Young animals were preferred to old ones because they are “functional monogastrics” and so the bioavailability of the RLE would not be affected by the ruminal or intestinal microbiota. After colostrum intake, all kids homogenous for age (4 days ± 12 h) and weight (3.1 ± 0.2 g), were divided into two throughout the experimental period, which lasted 40 days: Everyday animals in Group RLE (anthocyanins; *n* = 30) received an RLE extract (90 mg/kg of live weight) as oral food additive (see below), while those in Group CON (control; *n* = 30) received saline. Kids were weighed daily to record the average weight gain, and RLE extract was mixed with water to obtain a cream [12], which was then administered through a syringe per os. Animals were maintained in single boxes, where they received milk throughout the trial (1kg/die) three times a day and 150 g kids starter (20.5% crude protein DM, 1.8% fat DM, 25% crude fiber DM 150 g) and alfalfa hay (18.8% crude protein DM, 32.2% crude fiber DM) ad libitum from day 25 on. Feed intake was determined daily from the unconsumed feed before the next feeding. The amount and composition of refusals were used to evaluate the dry matter intake of kids.

Feed efficiency was calculated on all the experimental period (40 days) using the formula:

Feed efficiency = DM intake/weight gain.(1)

### 2.2. Relative Composition of Red Orange and Lemon Extract (RLE)

The standardized dry powder phytoextract rich in bioflavonoids (flavanones and anthocyanins) and other polyphenols was obtained by a patented extraction process (Italian Patent No. 102017000057761) from red orange and lemon processing wastes (Red orange and Lemon Extract, here named RLE). The standardized extract was created at CREA—Research Centre for Olive, Fruit and Citrus Crops (CREA-OFA, Acireale, Italy), for research purposes only. Further information about the identification and relative concentrations of individual flavanones and anthocyanins are already described in previous studies, and performed by HPLC-PDA-ESI/MS^n^ analysis [18,20]. The chemical composition of RLE is shown in Table 1.

### 2.3. Slaughter Procedures and Carcass Measurements

Kids were transported and slaughtered at a European Community-approved abattoir in compliance with European Community laws on Animal Welfare in transport (1/2005EC) and the European Community regulation on Animal Welfare for the slaughter of commercial animals (1099/2009EC).

The non-carcass components (head, skin, feet, lungs, trachea, heart, liver, spleen, gastro-intestinal tracts, and testicles) were removed, and the warm carcass weight of each kid was recorded. Warm dressing percentage was calculated with the formula WW/SW (WW = warm carcass weight; SW = slaughtering weight). The carcasses were chilled at 4 °C for 24 h, and the cold dressing percentages were assessed according to the formula CW/SW (CW = cold carcass weight). Carcass measurements were recorded, and according to Yakan et al. [11], some carcass indexes were calculated as: Length (from the caudal edge of the last sacral vertebra to the dorso-cranial edge of the atlas), internal carcass length (length from the cranial edge of the symphysis pubis to the cranial edge of the first rib), leg length (length from the symphysis pubis to the tarsal-metatarsal joint), chest circumference (circumference measurement of the chest at the widest rib area), chest width (widest chest measurement between left and right side at the rib area), leg compactness (leg weight/leg length; kg/m) and carcass compactness (cold carcass weight/carcass length; kg/m). Samples (2 mg) of the liver, kidney, and small intestine were removed from each animal and were put immediately at –20°C until oxidative assay determination.

The pH was recorded at the slaughtering, 1h and 24 h postmortem with a portable pH meter with glass electrode shaped to easily penetrate meat (Carlo Erba pH 710, Carlo Erba Reagenti, Milano, Italy). At each measurement, the pH meter was previously automatically calibrated for muscle temperature using standard solutions with 4 and 7 pH values (Crison, Lainate, Italy).

### 2.4. Morphological Analyses

Samples of the duodenum (4 cm removed from the pyloric sphincter) were collected from the two animal groups. The duodenum constitutes the first site of nutrient absorption, and morpho-physiological changes of glyconiugates can be more easily appreciated in response to dietary supplementation and/or changes. Upon collection, samples were treated for morphological analyses and then stained with hematoxylin and eosin. Carbohydrate characterization was performed by staining with Periodic acid–Schiff (PAS) to evidence glycogen, Alcian blue (AB), and PAS to distinguish neutral from acid mucins; Alcian blue pH 2.5 to identify acid mucins. All sections were observed under a Leica DM6B (Leica, Wetzlar, 206 Germany), and images were processed by LAS X software (Leica, Wetzlar, 206 Germany).

### 2.5. Chemicals and Reagents

All reagents were provided by Sigma-Aldrich (Milan, Italy; Lipid Peroxidation (MDA) Assay Kit, Item No. MAK085; Catalase Assay Kit, Item No. CAT100; Glutathione Assay Kit, Item No. 38185; SOD Assay Kit, Item No.19160; the standardized RLE was obtained by CREA-OFA (Acireale, Italy).

### 2.6. Meat Sampling and Analysis

The Longissimus thoracis et lumborum muscle was sampled (from the 1st thoracic to the 5th lumbar vertebra). It was cut into three parts, each one randomly assigned to one of the three experimental storage days: 1, 3, and 7. All the further analysis were performed on it. This muscle was conserved for seven days at a chilling temperature of 4 °C.

### 2.7. Chemical Composition

The chemical composition was analyzed only on the first aging day (day 1 after muscle sampling). Muscle samples were cleaned, and the epimysium was removed. To obtain a homogeneous mass, the muscle was triturated in a domestic blender. The moisture was determined by the formula IW-DW (IW= initial weight of the sample; DW= dried weight of the sample) at 105 °C for 24 h in the oven. The protein content was calculated according to ISO937:1978 [21], intramuscular fat (IMF) content according to ISO1443:1973 [22], and ash following ISO 936:1998 [23] protocols. Each sample was homogenized with a mixture of chloroform and methanol (1:2, *vol*/*vol*) solution for the extraction of total lipids from IMF [24].

### 2.8. Water Holding Capacity, Cooking Loss, and Post Thawing Loss

Water-holding capacity (WHC) was calculated using the centrifugation method, in duplicate, as described by De Palo et al. [25], the cooking loss was determined as described by De Palo et al. [26]. And the post thawing losses were calculated as described by De Palo et al. [27].

### 2.9. Meat Texture Profile Analysis

The Warner–Bratzler shear force (WBSF) was analyzed as described by De Palo et al. [27]. The texture profile analysis was performed at room temperature using a TA-XT2 texture analyzer (Stable Micro Systems, Godalming, UK). From each sample, a 1.5 cm height and 2 cm diameter cylinder was prepared from each sample. A double compression cycle test was performed up to 50% compression of the original portion height with an aluminum cylinder probe of 2 cm diameter. The two compression cycles were divided by 5 s of pause. Force–time deformation curves were obtained with a 25 kg load cell applied at a cross head speed of 2 mm/s. The following parameters were quantified on the surface of the muscle: Hardness (the maximum force of the first compression cycle required to compress the sample, N), adhesiveness (the negative area under the abscissa after the first compression, N_s), springiness (the ability of the sample to recover its original form after the deforming force was removed, cm), cohesiveness (the extent to which the sample could be deformed prior to rupture, dimensionless), chewiness (the work required to masticate a solid food before swallowing, J).

### 2.10. Colorimetric Analysis

The surface meat color was determined according to the CIE L*, a*, b* [28] color system using a Minolta CR-300 colorimeter (light source D65; Minolta Camera Co. Ltd., Osaka, Japan). Reflectance measurements were collected from a 0° viewing angle with an A-pulsed xenon arc lamp with a reading surface of 8 mm diameter. For each day, three measurements were performed on three different points as described by De Palo et al. [29]. The a* and b* values were used to determine chroma (C*) = (a^2^ + b^2^)^1/2^ and hue (radians, H_) = tan^–1^ (b/a) according to Maggiolino et al. [4].

### 2.11. Thiobarbituric Acid Reactive Substances, Protein Carbonyls, and Hydroperoxides Analyses

For each meat piece randomly assigned at each aging time, a minced sample (5 g) was placed in a 50-mL test tube and homogenized with 15 mL deionized distilled water (DDW). The thiobarbituric acid reactive substances (TBARS) determination was performed, according to Buege and Aust [30]. The hydroperoxides were determined according to De Palo et al. [31] method. Protein oxidation was calculated as reported by Tokur et al. [32].

### 2.12. Fatty Acids Methyl Ester (FAME) Analyses

The FAME were prepared by transesterification of the lipid extract, as described by De Palo et al. [33], using methanol in the presence of 3% hydrochloric acid in methanol (*vol*/*vol*). Then, fatty acid (FA) were determined with a Trace GC Thermo Quest Gas Chromatograph (Thermo Electron, Rodano, Milan, Italy) equipped with a flame ionization detector, after their esterification with methanol in the presence of 3% hydrochloric acid in methanol (*vol*/*vol*). The derivatives were separated on a capillary column (Supelco SP-2380 fused-silica column, 60-m length, 0.25-mm internal diameter and 0.20-mm film thickness; Sigma-Aldrich, St Louis, MO, USA). Injector and detector temperatures were held at 260 °C. Column oven program temperatures were as follows: T1 = 80 °C, hold 1 min; T2 = 150 °C ramp at 15 °C/min, hold 2 min; T3 = 220 °C ramp at 5 °C/min, hold 2 min; and T4 = 250 °C ramp at 15 °C/min, hold 5 min. The flow rate of the carrier gas (helium) was set at 0.8 mL/min. Identification of FAME was based on the retention times of reference compounds (Sigma-Aldrich, St Louis, MO, USA) and mass spectrometry. The FA composition was expressed as the percentage of total FAME (SupelcoTM 37 Component FAME Mix, Catalog Number 47885-U, Sigma-Aldrich). Nutritional implications were assessed by calculating the amount of saturated fatty acids (SFA), monounsaturated fatty acids (MUFA), polyunsaturated fatty acids (PUFA), n-3, and n-6 FA, as well as the PUFA:SFA and the n-6:n-3 ratios. Finally, both the atherogenic index (AI) and thrombogenic index were calculated according to the following formulas [34]:

Atherogenic index (AI) = (C12:0 + 4 × C14:0 + C16:0)/[ΣMUFA + ΣPUFA (n-6) and (n-3)](2)

Thrombogenic index (TI) = (C14:0 + C16:0 + C18:0)/[0.5ΣMUFA + 0.5ΣPUFA (n-6) + 3ΣPUFA(n-3) + (n-6)/(n-3)](3)

### 2.13. Malondialdheyde (MDA) Assay

Lipid peroxidation was determined by assaying the MDA levels according to Ohkawa et al. [35]. It was determined by the reaction of MDA with thiobarbituric acid (TBA) to form a colorimetric (532 nm) product, proportional to the MDA present. Kidney, liver, and intestine of animals treated or not treated for all the experimental period with RLE were homogenized on ice with MDA Lysis Buffer. To form the MDA-TBA adduct, a TBA solution was added to each sample and incubated al 95 °C for 60 min. Then, each reaction mixture was placed into a 96 well plate to measure the absorbance at 532 nm. Results were expressed as nanomoles/mg of protein.

### 2.14. Nitrite and Nitrate Assay

The nitrite (NO_2_) and nitrate (NO_3_) production, stable metabolites of nitrous oxide (NO) production, was determined in the supernatant of the kidney, liver, and intestine by Griess reagent as described by Ciarcia et al. [36] and data were expressed as picomoles of nitrite for mg of proteins.

### 2.15. Oxidative Stress Markers

The activity of Superoxide dismutase (SOD), Catalase (CAT), and Glutathione peroxidase (GPx) in samples was determined by using a spectrophotometer at 450 nm, 520 nm, and 412 nm, respectively, according to previous studies [37,38,39]. Samples from all groups were collected at the end of treatment and treated according to methods described by Tateo et al. [40].

### 2.16. Blood Parameters

All animals underwent jugular vein blood sampling, at the beginning (day 0), in the middle (day 20), and at the end (day 40) of RLE treatment. Two EDTA (K3) vacutainer tubes and two heparinized tubes were used for each animal to assess plasma TBARS, Hydroperoxides, Protein Carbonyl, Superoxide dismutase, catalase, and glutathione peroxidase oxidative parameters. Plasma was obtained by centrifugation at 1800 g for 20 min and stored at −80 °C until analysis. The TBARS were measured fluorometrically according to Gondim et al. [41] by adding 100 mL of plasma to a 0.37% thiobarbituric acid solution. Plasma reactive carbonyl derivative (RCD) levels were measured according to Faure and Lafond [42]. The RCD levels were determined by carbonyl reagent 2,4-Dinitrophenylhydrazine (DNPH). Plasma (200 mL) was mixed with 1 mL water and 2 mL 20% trichloroacetic acid and centrifuged at 1000× *g* for 10 min. The pellet was resuspended in 1 mL of 10 mmol/L DNPH and incubated for 60 min at 37.8 °C. For control, 1 mL of 1 mol/L hydrochloric acid was used instead of DNPH. Subsequently, 1 mL of 20% trichloroacetic acid was added, and the sample was centrifuged at 1000× *g* for 10 min. The pellet was washed with 1:1 ethanolethyl acetate solution and centrifuged at 1000× *g* for 10 min. The pellet was mixed with 1 mL of 6 mol/L guanidine (diluted in 20 mmol/L dihydrogenphosphate at pH 2.3). Finally, the sample was incubated for 40 min at 37.8 °C. The absorbance was measured at 380 nm.

Hydroperoxides were analyzed, according Sodergren et al. [43]. Aliquots (90 mL) of plasma were transferred into eight microcentrifuge vials (1.5 mL). Ten microliters of 10 mM triphenylphosphine (TPP) in methanol were added to four of the vials to reduce ROOHs, thereby generating a quadruplicate of blanks. Methanol (10 mL) was added to the remaining four vials to produce a quadruplicate of test samples. All vials were then vortexed and incubated at room temperature for 30 min prior to the addition of 900 mL of FOX2 reagent. After mixing, the samples were incubated at room temperature for 30 min. The vials were centrifuged at 2400× *g* for 10 min with a swing-out rotor (Hettich Rotenta/RP centrifuge, Hettich-Zentrifugen, Tuttlingen, Germany). The absorbance of the supernatant was measured at 560 nm using an Ultraspec 2000 spectrophotometer (Pharmacia Biotech, Uppsala, Sweden). ROOH concentration in the plasma samples was calculated using the mean absorbance difference between quadruplicates of test samples and blank samples.

The SOD (EC 1.15.1.1) activity was examined according to Misra [44], the CAT (EC 1.11.1.6) activity was assayed by the method of Clairborne [45], and the GPx (EC1.11.1.9.) activity was measured according to Gunzler [46] as described by Tateo et al. [40].

### 2.17. Statistical Analysis

The data set was tested for normal distribution and variance homogeneity (Shapiro-Wilk). Each kid represented an experimental unit. The data sets of live performances, slaughtering performances, carcass measurements, indexes, and meat chemical composition were subjected to analysis of variance (ANOVA) using the general linear models (GLM) by SAS software [47], according to the following model:

y_i_ = μ + A_i_ + ε_ij,_ (4)

where y_ijk_ represents all the previous cited patterns as dependent variables; μ is the overall mean; A was the effect of the ith inclusion of the anthocyanin in the diet (I = 1, 2) and ε_ij_ was the error term.

The colorimetric, texture, oxidative parameters, and fatty acid profile were analyzed using the MIXED procedure of SAS [47] with repeated measures, according to the following model:

y_ijk_ = μ + α_i_ + A_j_ + T_k_ + (A × T)_jk_ + ε_ijkl,_ (5)

where y_ijk_ represented all the previous cited patterns as dependent variables; μ was the overall mean; α_i_ was the constant; A was the effect of the jth inclusion of the anthocyanin in the diet (j = 1, 2), T was the effect of the kth time (k = 1, …, 3), A × T was the effect of the interaction of the jth anthocyanin inclusion in the diet and kth time (1,…, 6), and ε_ijkl_ was the error term. When not significant, the binary interaction was dropped from the model. A Tukey test was applied to evaluate the differences according to the time. The significance was set at *p* < 0.05, and the results were expressed as quadratic means and mean standard error.

## 3. Results

### 3.1. Effects of Feeding on Growth Performances

No differences were recorded in terms of dry matter intake, initial and final body weight in both groups. However, a higher average daily gain and a better feed efficiency were reported in Group RLE compared to Group CON (Table 2)

### 3.2. Effects of Feeding on Animal Carcasses

The only difference was recorded for the cold carcass weight (kg) between groups (*p* = 0.01). No differences in terms of warm carcass weight (kg), pH measured at different times, and percentage of warm and cold dressing between RLE and CON groups were reported (Table 3). Moreover, no differences were recorded in carcass measurements, compactness indices, and cuts indices (Supplementary Table S1).

### 3.3. Glyco-Histochemistry of Duodenum

**Figure 1:**
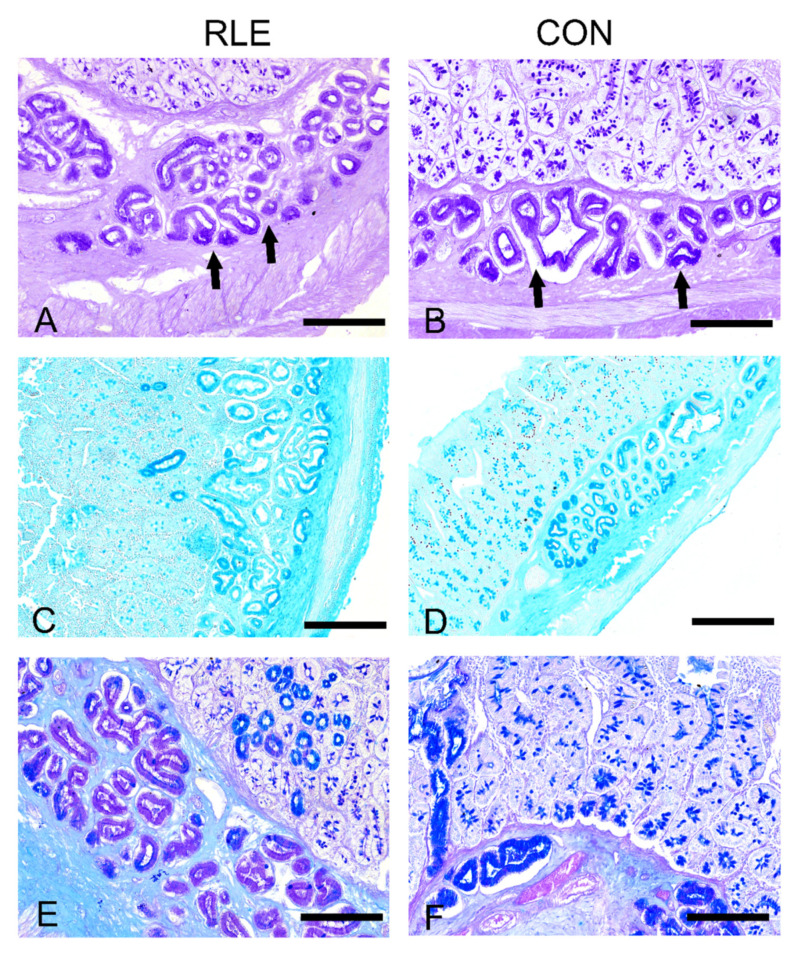
The histochemical response of kids’ duodenum secretory structures. (**A**,**B**). PAS (periodic acid–Schiff) staining showing less strong staining of Brunner’s glands in Group CON compared to Group RLE. Arrows indicate Brunner glands. (**C**,**D**). Alcian blue staining (pH 2.5) displaying a weaker histochemical response in goblet cells and Brunner’s glands of the control group compared to Group RLE. (**E**,**F**). Alcian-PAS staining depicting much less intense Alcian blue staining in the globet cells and Brunner’s glands of CON animals compared to RLE animals. Scale bars: (**A**,**B**,**E**,**F**) = 100 µm; (**C**,**D**) = 50 µm.

The appearance of the duodenum of the two experimental groups did not reveal morphological differences. We observed small intestine villi covered by a simple columnar epithelium, with goblet cells and simple tubular glands; at the base of epithelial villi, the crypts of Lieberkuhn are visible. The submucosa is predominantly occupied by Brunner’s glands. We observed PAS staining in control and treated animals in the globet cells, the crypts of Lieberkuhn, and Brunner’s glands (Figure 1A,B). In treated animals, the staining in the Brunner’s glands appeared weaker (Figure 1B). Similarly, goblet cells and duodenal submucosal glands were stained with Alcian blue (Figure 1C,D) at pH 2.5 and Alcian-Pas (Figure 1E,F) in both experimental groups. This pattern was confirmed when conducting Alcian-PAS staining, which revealed weaker staining either in the globet cells and Brunner’s glands in Group RLE compared to CON animals (Figure 1E,F).

### 3.4. Chemical Composition

No differences were recorded regarding meat chemical composition between groups in terms of moisture, proteins, fats, and ashes, respectively, in Group RLE and CON (Supplementary Table S2).

### 3.5. Water Holding Capacity, Cooking Loss, and Post Thawing Loss

Data regarding water holding capacity, cooking loss, and post thawing loss were reported in Table 4. The pH and the water holding capacity of samples in both groups decreased over time, from day 1 to day 7 (*p* < 0.01), but no differences were recorded between groups. The same trend was also observed for the sheer force and chewiness (*p* < 0.01). Moreover, the juiciness decreased in both groups over time (*p* < 0.01), and on day 7, was significantly higher in Group RLE compared to Group CON (*p* < 0.01). Regarding cooking loss percentage, it increased over time in Group CON (*p* < 0.01), becoming significantly different on day 7 compared to Group RLE (*p* < 0.05). No differences were recorded for all the other parameters.

### 3.6. Colorimetric Analysis, TBARS, Protein Carbonyls, and Hydroperoxides in Meat

Data regarding meat color and oxidative parameters are presented in Table 5. While lightness, redness, and chroma changed only over time (*p* < 0.01), the yellowness and the hue angle decreased over time (*p* < 0.01), and on day 7, were statistically different between Group RLE and Group CON (*p* < 0.05 for yellowness and *p* < 0.01 for hue angle). The TBARS increased over time (*p* < 0.01), and already on day 3, were statistically different between groups (*p* < 0.01). This result was confirmed on day 7. The hydroperoxides followed the same pattern of the TBARS, but become significantly different between groups only on day 7 (*p* < 0.01). The protein carbonyls did not differ between groups, but only over time (*p* < 0.01).

### 3.7. Fatty Acid Assay

The effect of RLE and aging time on the fatty acid profile of kids’ meat was reported in Table 6. The n3 and n6 values were always significantly higher in Group RLE compared to Group CON, since day 1 (*p* < 0.05 for n3 and *p* < 0.01 for n6), and they did not differ over time. Similarly, also the SFA, MUFA, PUFA, and UFA values, together with their ratio (SFA/PUFA and SFA/UFA), were always significantly different only between groups (*p* < 0.01) and not throughout time. Group RLE showed a lower concentration of SFA and a higher concentration of UFA, and consequently of MUFA and PUFA since day 1, compared to Group CON. The same pattern was also recorded in IA and IT, which were significantly lower (*p* < 0.01) in Group RLE, compared to Group CON throughout the experimental time.

### 3.8. Lipid Peroxidation

**Figure 2:**
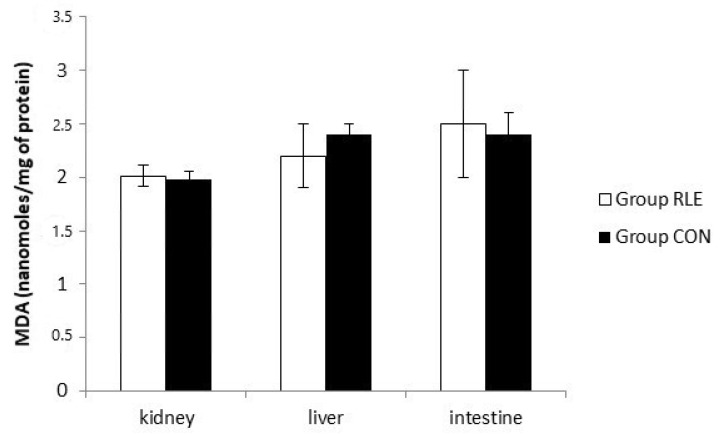
Effects of red orange and lemon extract (RLE) on lipid peroxidation measured by malondialdehyde (MDA) test at the end of treatment. Group RLE (RLE treated group), Group CON (control) untreated group.  Results are expressed as mean ± SD.

Figure 2 shows the lipid peroxidation result. MDA levels, expressed as nmol/mg of proteins, in all tissues did not significantly increased in Group RLE with respect to Group CON.

### 3.9. Nitrite and Nitrate Assay

**Figure 3:**
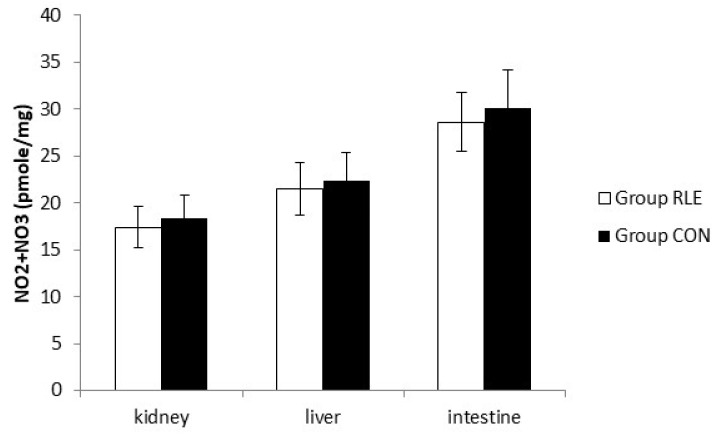
Effect of red orange and lemon extract (RLE) on nitric oxide (NO) production from kidney, liver, and intestine cell at the end of treatment measured by Griess reagent. Group RLE (RLE treated group); Group CON (control). Data are expressed as mean ± standard deviation (SD).

As shown in Figure 3, there was no significant difference in NO production at the end of treatment.

### 3.10. Antioxidant Enzymes SOD, CAT, GPx Activity

No significant difference in the SOD activity was observed in the renal tissue about Group RLE and Group CON at the end of treatment. The same result was observed in the liver and intestine (Table 7). The CAT activity of all tissues did not change in Group RLE with respect to Group CON at the end of treatment (Table 7). Moreover, RLE did not affect GPx activity of the kidney, liver, and intestine (Table 7).

### 3.11. Blood Antioxidant Profile

The in vivo blood parameters were reported in Table 8. The superoxide dismutase, catalase, and glutathione peroxidase changed over time and groups. In particular, they increased in both groups, from day 1 (*p* < 0.01), but they were significantly higher in Group RLE compared to Group CON, starting from day 20 (*p* < 0.01). No differences were recorded for all the other parameters.

## 4. Discussion

This study evaluated the effects of RLE supplementation, rich in bioflavonoids, in Saanen kids. The utilization of byproducts as feed for livestock has been largely investigated in the last years [48,49]. “From waste to resource” is considered a basic concept of the circular economy for several reasons. Their use as feed for livestock would reduce either the costs of animal productions or land utilization for feeding livestock, simultaneously increasing the nutraceutical properties of animal-derived food [50]. Furthermore, it is worth pointing out that waste disposal is one of the main causes of environmental pollution, representing a serious item in terms of sustainability in several production chains, because of their content in organic material [48]. However, the latter is an important source of many bioactive compounds, such as sugars, minerals, vitamins, polyphenols, etc. The chance of re-cycling these materials and proper use as feed is an unmissable challenge for the livestock system.

Although the antioxidant properties of anthocyanins are largely known [12], no studies have been carried out to our knowledge in goat meat production. The effect of plant extracts administration on both in vivo performance in kids is a debated topic: Some authors report positive effects [51,52], while others report only no adverse effects [5,6,10]. This could be due to both the large variability in the composition of the extracts and the scarce information about the bioavailability of phenolic compounds in ruminants and monogastric animals. Indeed, even though it is known that bacterial rumen population can change after polyphenols administration, modifying fermentation and absorption [53], no information are available on “functional monogastrics”, as kids. It can be hypothesized that, as in non-ruminant species, the bioavailability of these substances is affected by their chemical characteristics and the intestinal microbiota [53]. Furthermore, the amount of RLE extract that was administered (90 mg/kg) was defined according to some studies performed in monogastric animals [18]: It cannot be ruled out that in the last 10–15 days of the trial, partial development of the rumen may have further influenced their absorption and metabolization affecting the antioxidant profile of kids’ meat.

In our study, no effects of the dietary treatment with RLE were recorded on daily average gain and carcass characteristics, such as carcass weight and conformation, carcass compactness indexes, and pH, during aging. However, better feed efficiency was reported in Group RLE compared to Group CON, which, in turn, has led to a higher average daily gain. Moreover, no differences were recorded between the two groups on meat chemical composition in terms of moisture, proteins, fat, and ashes. According to other reports [11,54,55], and in particular, to Cimmino et al. [10], who also experienced a supplementation of a plant extract used as a feed additive in growing kids, a similar meat chemical composition was observed in both groups. However, Lobo et al., 2020 [56], who studied the inclusion of Yerba Mate (*Ilex paraguariensis*) extract in the diet of growing lambs, demonstrated that the inclusion of up to 2% of this extract improved dry matter intake, total weight gain, and daily weight gain. Our findings report no differences in pH, shear force, chewiness, and water holding capacity of samples between groups. A similar trend was observed for these parameters during aging, showing a reduction independently from anthocyanins administration. On the contrary, juiciness decreased in both groups throughout the trial, but it was significantly higher in Group RLE compared to Group CON on day 7. An opposite trend was reported for the cooking loss percentage, which increased over time in Group CON. Yakan et al. [11] reported that meat produced from goats both grazing or fed concentrate with Vit E supplementation was juicier and tenderer compared to meat produced by animal fed commercial concentrate feed. It has been reported [57] that the amount of oleic acid could influence tenderness by affecting the melting point, improving tenderness and juiciness in meat. Indeed, in our experiment, Group RLE showed a higher amount of oleic acid compared to Group CON, supporting our results.

Meat color is the parameter that most influences consumers’ punctual purchasing decisions, because it is the only trait they can evaluate at the point of sale [4,58]. Muscle color has significant importance for consumers in suckling lamb and kid production, whose carcasses should be pale or pink. Regardless of dietary treatment, aging decrease most of the colorimetric parameters in both groups, except for lightness that increased on day 7. This could be easily explained by the passage of water from the intracellular to the extracellular muscle fiber during aging, due to their breakdown that increases lightness values [31]. In our study, yellowness and hue angle decreased less in Group RLE on day 7, compared to Group CON. Yellowness is strictly linked to the meat’s oxidative stability [7], while hue angle is a good descriptor of meat browning [59]. We can speculate that the treatment with RLE could slightly improve meat color stability during aging, even if this modification may also depend on other factors, such as feed content or feeding regimen.

Several studies showed how using antioxidant substances, improves meat quality by decreasing lipid peroxidation and the formation of DNA additives, by inhibiting the formation of MDA, an aldehyde commonly utilized as a marker of secondary lipid oxidation in meat [4,60]. The administration of RLE positively affects the oxidative stability of the meat, as demonstrated by the reduction of MDA. Indeed, although in our experiment we observed an increased production of lipid and protein oxidation catabolites in both groups, due to aging time, lower meat TBARS values, and hydroperoxide production, were recorded in Group RLE, compared to CON. In any case, no differences were observed in TBARS and Hydroperoxide plasma levels when RLE was added to the diet. We have also investigated the lipid peroxidation production by MDA assay in the liver, kidney, and intestine, without highlighting significant differences between groups. Similarly, no significant differences in the SOD, CAT, and GPX activities were observed in these tissues between Group RLE and CON at the end of treatment.

However, in plasma, higher concentrations of SOD, GPX, and CAT were found in Group RLE compared to Group CON. These results are in agreement with previous studies, in which using antioxidant bioactive substances resulted in increased enzyme antioxidant activity in both kids’ meat [5,61] and plasma [62,63]. Although there is a tendential increase in the antioxidant activities of SOD, CAT, and GPx in the Group RLE with respect to Group CON, no significant difference was observed in the kidney, intestine, and liver of both groups. Probably this result is related to the low number of samples, and more investigations will be necessary.

In addition, at intestinal level, the increased goblet cells reactivity to AB pH 2.5 of animals fed with RLE extract, rich in anthocyanin, suggests that the supplemented feed determines a higher production of acidic carboxylated residues than neutral carbohydrates, which may have a direct influence on nutrient absorption and microbial intestinal colonization and action [64,65]. Remarkably, at the gastrointestinal level, RLE used as feed additives in goats determined an increase of NPY in the enteroendocrine cells of the abomasum and pancreas, but not in the duodenum [66].

It is already known that diet plays a pivotal role in the composition and modification of fatty acid that, in turn, depends on the rumen microbial population. Indeed, different diets could influence rumen population activity and the hydrogenation of different UFA [67]. In our study the inclusion of anthocyanins influenced the fatty acid profile of the meat. Meat in Group RLE was characterized by lower SFA and both higher MUFA and PUFA concentrations, compared to Group CON. In particular, we found lower concentrations of some saturated fatty acids, as well as palmitic (C16:0) and stearic (C18:0) acids and higher concentrations of unsaturated fatty acid, such as myristoleic (C14:1), oleic (C18:1), and linoleic (C18:2 n6) acids in Group RLE compared to Group CON. The oleic acid was the most represented acid within the fatty acid composition followed by palmitic and stearic: These results align with different studies made on goat meat [10,11,68]. The addition of the anthocyanins led to a reduction of n3 levels and an increasing level of n6, during aging in both groups. In any case, the n3/n6 ratio was not different between groups: It has also to be considered that the effect of anthocyanins depends on dosage or on the timing for meat fat oxidation and fatty acid composition. However, lower TI and AI were recorded in Group RLE since day 0 compared to Group CON, and this may suggest that the addition of the RLE in the diet could lead to a decreased risk of cardio-metabolic disease for consumers [69]. Moreover, TI and AI levels are higher below the standards (respectively < 1.3 and < 1.01) [70,71], suggesting that anthocyanin administration may improve meat quality.

## 5. Conclusions

In conclusion, the addition of 90 mg/kg of RLE to kids’ diet regimen did not affect in vivo growth performance and on meat chemical composition. However, RLE inclusion improved juiciness and reduced color deterioration and cooking loss, making the kids’ meat more attractive to consumers at first sight. Finally, the quality of the meat was positively influenced by delaying lipid oxidation, and the consequent rancidity, and had a better fatty acid profile—resulting in healthier meat for human consumption.

## Figures and Tables

**Table 1:** Chemical composition of red orange and lemon extract (RLE) used as an oral food supplement in this study.

Class of Compounds	Relative Composition (%)
Total flavanones (as hesperidin equivalents)	15.91 ± 0.01
Total anthocyanins (as cyanidin 3-glucoside equivalents)	2.66 ± 0.01

**Table 2:** Effect of dietary treatment on growth performance in terms of live weight gains of kids, dry matter intake, and feed efficiency.

Parameter	Group		SEM	*p*-Value
	RLE	CON		
Dry matter intake, g/d	164.35	171.25	11.35	0.3347
Initial body weight, kg	3.18	3.33	0.11	0.2541
Final body weight, kg	7.87	7.55	0.24	0.2657
Average daily gain, g/d	119.75	107.25	7.26	0.0221
Feed efficiency	1.26	1.55	0.07	0.0005

SEM, Standard error of the means; RLE, red orange and lemon extract group; CON, control group; SEM, standard error of the means.

**Table 3:** Effect of including RLE in the diet on kids slaughtering performances in Group RLE (RLE treated group); Group CON (control).

	Group RLE	Group CON	SEM	*p* Value
Live weight at slaughter (kg)	8.25	7.87	0.60	0.67
Warm carcass weight (kg)	4.51	4.40	0.35	0.83
Cold carcass weight (kg)	4.44	4.39	0.34	0.91
pH 0h	7.25	7.26	0.05	0.89
pH 1h	7.14	7.19	0.04	0.85
pH 24h	6.92	6.92	0.06	0.96
Warm dressing (%)	60.10	61.44	0.51	0.09
Cold dressing (%)	59.20	61.47	0.55	0.01
Skin and limbs incidence (%)	9.12	10.25	1.20	0.31
Liver, heart and lungs incidence (%)	4.85	5.12	0.61	0.59

SEM, Standard error of the means; RLE, red orange and lemon extract group; CON, control group; SEM, standard error of the means.

**Table 4:** Effect of including anthocyanins in the diet and of aging time on kid’s meat rheological parameters and texture profile analysis. Group RLE (RLE treated group); Group CON (control).

Group	Day 1	Day 3	Day 7	SEM		Significance	
	Ph				Group	Aging	GroupxAging
RLE	6.91 ^A^	6.86 ^A^	6.59 ^B^	0.03	0.4881	<0.0001	0.3937
CON	6.94 ^A^	6.83 ^A^	6.55 ^B^				
	Water holding capacity (%)						
RLE	89.33 ^a^	87.81	86.12 ^b^	9.76	0.9086	0.0021	0.8927
CON	89.29 ^a^	87.38	85.92 ^b^				
	Cooking loss (%)						
RLE	35.86	38.17	39.10 ^x^	1.02	0.0001	0.0004	0.5507
CON	38.30 ^A^	41.41	43.76 ^B, y^				
	Post Thawing loss (%)						
RLE	5.87	6.47	7.62	0.62	0.8030	0.0214	0.9927
CON	5.95	6.68	7.71				
	Shear Force (N)						
RLE	23.66 ^Aa^	20.08 ^b^	18.59 ^B^	0.86	0.0004	<0.0001	0.9573
CON	26.29 ^A^	23.02	21.02 ^B^				
	Adhesiveness (−N × s)						
RLE	0.31	0.32	0.34	0.02	0.3408	0.7278	0.7858
CON	0.30	0.32	0.30				
	Springiness (cm)						
RLE	0.90	0.84	0.80	0.04	0.3468	0.4659	0.3056
CON	0.82	0.81	0.83				
	Cohesiveness (-)						
RLE	0.46	0.45	0.46 ^x^	0.02	0.0023	0.2110	0.0658
CON	0.45	0.40	0.39 ^y^				
	Juicyness (-)						
RLE	0.59 ^A^	0.51 ^B^	0.47 ^B, X^	0.01	<0.0001	<0.0001	0.0468
CON	0.58 ^A^	0.48 ^B^	0.40 ^C, Y^				
	Chewiness (J × 10^−2^)						
RLE	4.29 ^A^	3.80	3.39 ^B^	0.13	0.0002	<0.0001	0.7972
CON	4.62 ^A^	4.30	3.87 ^B^				

SEM, Standard error of the means. Different letters in the same row show statistical differences: A, B, C = *p* <0.01; a, b = *p* < 0.05. Different letters in the same column, for each parameter, show statistical differences: X, Y = *p* < 0.01; x, y = *p* < 0.05.

**Table 5:** Effect of including anthocyanins in the diet and of aging time on kid’s meat color and oxidative parameters.

Group	Day 1	Day 3	Day 7	SEM	Significance		
	Lightness				Group	Aging	Group × Aging
RLE	47.35 ^A^	46.86	52.46 ^B^	0.52	0.0270	<0.0001	0.5123
CON	45.70 ^A^	46.36	51.74 ^B^				
	Yellowness						
RLE	2.26 ^A^	1.69 ^B^	0.90 ^C, x^	0.09	<0.0001	<0.0001	0.7248
CON	2.00 ^A^	1.31 ^B^	0.50 ^C, y^				
	Redness						
RLE	10.22 ^A^	10.26	8.83 ^B^	0.26	0.1407	<0.0001	0.8362
CON	11.70 ^Aa^	10.41 ^b^	9.19 ^Bc^				
	Hue						
RLE	0.21 ^A^	0.16 ^B, x^	0.10 ^B, X^	0.01	<0.0001	<0.0001	0.6351
CON	0.17 ^A^	0.12 ^B, y^	0.05 ^C, Y^				
	Chroma						
RLE	65.60 ^Aa^	54.32 ^Ab^	39.89 ^B^	2.59	0.1570	<0.0001	0.7901
CON	70.32 ^A^	55.48 ^Ba^	43.12 ^Bb^				
	TBARS (mg MDA/kg)						
RLE	0.33 ^A^	0.56 ^X^	0.71 ^B, X^	0.08	<0.0001	<0.0001	0.0038
CON	0.36 ^A^	0.60 ^B, Y^	1.07 ^C, Y^				
	Hydroperoxides (µmol/g)						
RLE	0.33 ^A^	0.55 ^Ba^	0.70 ^Bb, X^	0.21	<0.0001	<0.0001	<0.0001
CON	0.36 ^A^	0.60 ^B^	1.07 ^C, Y^				
	Protein carbonyl (nmol DNPH/mg protein)						
RLE	3.24 ^A^	3.65	4.05 ^B^	0.16	0.5630	<0.0001	0.9745

SEM, Standard error of the means. RLE, red orange and lemon extract group; CON, control group. Different letters in the same row show statistical differences: A, B, C = *p* < 0.01; a, b, c = *p* < 0.05. Different letters in the same column, for each parameter, show statistical differences: X, Y = *p* < 0.01; x, y = *p* < 0.05.

**Table 6:** Effect of including RLE in the diet and of aging time on kid’s meat fatty acids profile and groups (expressed as % of total fatty acid methyl ester) atherogenic index and thrombogenic index. Group RLE (RLE treated group); Group CON (control).

	Group	Day 1	Day 3	Day 7	SEM	Significance		
C 8:0	RLE	1.41	1.45	1.48	0.03	0.1134	0.2441	0.9922
	CON	1.46	1.52	1.53				
C 10:0	RLE	0.61	0.50	0.57	0.04	0.0059	0.0208	0.8921
	CON	0.71	0.60	0.64				
C 12:0	RLE	0.42	0.36	0.38	0.04	0.0048	0.9683	0.2355
	CON	0.44	0.51	0.50				
C 12:1	RLE	0.06	0.04	0.04	0.01	<0.0001	0.8453	0.2296
	CON	0.05	0.05	0.06				
C 14:0	RLE	3.77	3.77	4.02	0.20	0.0009	0.0658	0.7420
	CON	4.78	4.56	4.35				
C 14:1	RLE	0.26 ^x^	0.22 ^x^	0.18 ^x^	0.03	<0.0001	0.7052	0.4790
	CON	0.25 ^y^	0.21 ^y^	0.23 ^y^				
C 15:0	RLE	0.33	0.38	0.40	0.03	0.6774	0.8788	0.6552
	CON	0.41	0.49	0.46				
C 16:0	RLE	20.92 ^x^	21.50	20.68 ^x^	0.42	<0.0001	0.4436	0.2572
	CON	22.84 ^y^	22.88	23.09 ^y^				
C 16:1	RLE	3.75	4.04	4.10	0.17	0.0854	0.0008	0.6213
	CON	4.84	4.29	4.12				
C 17:0	RLE	1.95	2.06	2.10	0.12	0.1126	0.6330	0.7680
	CON	2.12	2.03	2.09				
C 17:1	RLE	1.66	1.53	1.63	0.09	0.2726	0.2864	0.0051
	CON	1.51	1.43	1.51				
C 18:0	RLE	11.22 ^X^	11.32 ^x^	11.50 ^X^	0.31	0.6982	0.2132	0.4411
	CON	13.69 ^Y^	12.81 ^y^	13.18 ^Y^				
C 18:1	RLE	38.23 ^X^	37.60 ^X^	37.26 ^X^	0.48	0.4303	0.0824	0.7921
	CON	34.26 ^Y^	35.02 ^Y^	34.48 ^Y^				
C 18:2 n6	RLE	12.34 ^X^	12.67 ^X^	12.99 ^X^	0.21	0.0891	0.4357	0.9490
	CON	10.71 ^Y^	11.07 ^Y^	11.39 ^Y^				
C18:2(CLA) cis-9-trans-11	RLE	1.02	1.24	1.09	0.05	<0.0001	0.6102	0.2932
	CON	1.17	1.19	1.21				
C 18:3 n6	RLE	0.09	0.08	0.11	0.01	<0.0001	0.0102	0.9959
	CON	0.07	0.09	0.09				
C 18:3 n3	RLE	0.36	0.34	0.37	0.04	0.1680	0.1412	0.0641
	CON	0.33	0.31	0.34				
C 20:0	RLE	0.58	0.33	0.36	0.08	0.2407	0.6987	0.9994
	CON	0.72 ^a^	0.49	0.38 ^b^				
C 20:1	RLE	0.05	0.04	0.03	0.01	0.2302	0.0466	0.1207
	CON	0.03	0.04	0.03				
C 20:2 n6	RLE	0.04	0.05	0.03	0.01	0.2486	0.0797	0.4134
	CON	0.05	0.04	0.04				
C 20:4 n6	RLE	0.22	0.19	0.22	0.03	0.2478	0.2639	0.9585
	CON	0.19	0.15	0.20				
C 20:5 n3	RLE	0.48 ^X^	0.44 ^x^	0.42	0.02	<0.0001	0.1593	0.1109
	CON	0.39 ^Y^	0.38 ^y^	0.40				
C 22:0	RLE	0.27	0.26	0.26	0.01	0.9462	0.6542	0.1896
	CON	0.28	0.28	0.27				
C 22:1	RLE	0.04	0.04	0.04	0.01			
	CON	0.04	0.04	0.03				
C 22:5 n3	RLE	0.67 ^a^	0.58 ^b, x^	0.62 ^X^	0.02	<0.0001	<0.0001	0.0013
	CON	0.62 ^A^	0.48 ^B, y^	0.41 ^B, Y^				
C 22:6 n3	RLE	0.24	0.23	0.24	0.02	0.3247	0.5353	0.2582
	CON	0.20	0.23	0.24				
n6	RLE	12.70 ^X^	12.98 ^X^	13.36 ^X^	0.21	<0.0001	0.0101	0.9939
	CON	11.03 ^Y^	11.36 ^Y^	11.71 ^Y^				
n3	RLE	1.75 ^x^	1.54 ^x^	1.65 ^X^	0.05	<0.0001	0.0039	0.6333
	CON	1.53 ^y^	1.39 ^y^	1.37 ^Y^				
n6/n3	RLE	7.30	8.24	8.09	0.28	0.4256	0.0005	0.5816
	CON	7.29	8.29	8.61				
SFA	RLE	41.49 ^X^	41.94 ^X^	41.75 ^X^	0.50	<0.0001	0.6517	0.2045
	CON	47.47 ^Y^	46.17 ^Y^	46.47 ^Y^				
MUFA	RLE	44.06 ^X^	43.50 ^x^	43.29 ^X^	0.49	<0.0001	0.6811	0.2198
	CON	39.97 ^Y^	41.09 ^y^	40.44 ^Y^				
PUFA	RLE	14.44 ^X^	14.56 ^X^	15.01 ^X^	0.22	<0.0001	0.0512	0.9669
	CON	12.56 ^Y^	12.75 ^Y^	13.08 ^Y^				
UFA	RLE	58.51 ^X^	58.06 ^X^	58.30 ^X^	0.51	<0.0001	0.6391	0.2108
	CON	52.53 ^Y^	53.83 ^Y^	53.52 ^Y^				
SFA/PUFA	RLE	2.89 ^X^	2.88 ^X^	2.79 ^X^	0.07	<0.0001	0.0816	0.5065
	CON	3.80 ^Y^	3.63 ^Y^	3.56 ^Y^				
SFA/UFA	RLE	0.71 ^X^	0.72 ^X^	0.72 ^X^	0.02	<0.0001	0.5131	0.2059
	CON	0.91 ^Y^	0.86 ^Y^	0.87 ^Y^				
AI	RLE	0.62 ^X^	0.64 ^X^	0.64 ^X^	0.02	<0.0001	0.6573	0.1941
	CON	0.81 ^Y^	0.78 ^Y^	0.77 ^Y^				
TI	RLE	0.88 ^X^	0.88 ^X^	0.88 ^X^	0.02	<0.0001	0.2170	0.1327
	CON	1.11 ^Y^	1.04 ^Y^	1.05 ^Y^				

RLE, red orange and lemon extract group; CON, control group; SEM, standard error of the means; SFA, saturated fatty acids; MUFA, monounsaturated fatty acids; UFA, unsaturated fatty acids; AI, atherogenic index; TI, thrombogenic index. Different letters in the same column, for each parameter, show statistical differences: X, Y = *p* < 0.01; x, y = *p* < 0.05. Different letters in the same row show statistical differences: A, B = *p* < 0.01; a, b = *p* < 0.05.

**Table 7:** Effects of red orange and lemon extract (RLE) on SOD, CAT, and GPx activities in renal, liver, and intestine of experimental groups at the end of treatment.

	Group	Kidney	Liver	Intestine
SOD (U/mg)	RLE	143.8 ± 11.3	139.5 ± 22.2	131.6 ± 11.4
	CON	140.3 ± 11.1	141.1 ± 13.7	129.6 ± 12.8
CAT (U/mg)	RLE	137.1 ± 11.1	123.4 ± 12.4	129.0 ± 12.4
	CON	128.0 ± 10.1	122.5 ± 12.2	125.2 ± 14.1
GPx (U/mg)	RLE	38.4 ± 4.1	33.1 ± 3.1	37.4 ± 2.2
	CON	35.2 ± 2.9	32.8 ± 3.1	36.6 ± 2.5

RLE, red orange and lemon extract group; CON, control group; SEM, standard error of the means; TBARS, Thiobarbituric acid reactive substances; SOD, superoxide dismutase; CAT, catalase; GSPx, glutathione peroxidase. Data are expressed as mean ± standard deviation (SD).

**Table 8:** Effect of including RLE in the diet during the growing of the kid’s plasma TBARS, hydroperoxides, protein carbonyl, superoxide dismutase, catalase, and glutathione peroxidase oxidative parameters.

	Group	Day 1	Day 20	Day 40	SEM ^1^	Significance		
						Group	Age	Group × Age
TBARS (nmol/mL)	RLE	1.52	1.54	1.43	0.05	0.7558	0.4195	0.5791
	CON	1.48	1.49	1.48				
Hydroperoxides (mmol/mL)	RLE	5.73	6.07	6.61	0.25	0.6724	0.0671	0.3042
	CON	5.80	6.27	6.08				
Protein Carbonyl (mmol/L)	RLE	105.77	106.10	101.18	1.64	0.3988	0.1860	0.3200
	CON	103.83	102.94	102.85				
Superoxides dismutase (U/mg protein)	RLE	31.86 ^A^	45.66 ^B, X^	60.28 ^C, X^	0.42	<0.0001	<0.0001	<0.0001
	CON	31.35 ^A^	23.96 ^B, Y^	35.57 ^C, Y^				
Catalase (U/mg protein)	RLE	0.52 ^A^	0.92 ^B, X^	1.19 ^C, X^	0.01	<0.0001	<0.0001	<0.0001
	CON	0.53 ^A^	0.57 ^B, Y^	0.64 ^C, Y^				
Glutatione peroxidase (nmol NADPH ox/mg protein)	RLE	3.51 ^A^	7.19 ^B, X^	12.03 ^C, X^	0.10	<0.0001	<0.0001	<0.0001
	CON	3.47 ^A^	4.15 ^B, Y^	8.52 ^C, Y^				

^1^ Standard error of the means. Data are expressed as mean ± standard deviation (SD). Different letters in the same column, for each parameter, show statistical differences: X, Y = *P* < 0.01; x, y = *P* < 0.05. RLE, red orange and lemon extract group; CON, control group; TBARS, Thiobarbituric acid reactive substances. Different letters in the same row show statistical differences: A, B, C = *p* < 0.01; a, b, c = *p* < 0.05.

## Data Availability

Not applicable.

## Supplementary Materials

The following are available online at https://www.mdpi.com/2076-2615/11/3/809/s1, Table S1: Effect of including RLE in the diet on kids’ carcass measurements and cuts incidence. Group RLE (RLE treated group); Group CON (control), Table S2: Effect of including RLE in the diet on kids’ meat chemical composition (expressed as g/100 g of meat). Group RLE (RLE treated group); Group CON (control).
