# Supplementary material for: Productive Performance and Meat Characteristics of Kids Fed a Red Orange and Lemon Extract
Source: Animals (Basel). 2021 Mar 13;11(3):809. doi: 10.3390/ani11030809 (PMC7999896; doi:10.3390/ani11030809)
Supplement: Supplementary file 1 [file animals-11-00809-s001.pdf]

animals

Supplementary Material

Productive Performance and Meat Characteristics of Kids Fed a Red Orange and Lemon Extract

Angela Salzano [1,†], Sara Damiano [1,†], Livia D'Angelo [1], Gabriele Ballistreri [2], Salvatore Claps [3], Domenico Rufrano [3], Aristide Maggiolino [4,*], Gianluca Neglia [1], Pasquale De Palo [4] and Roberto Ciarcia [1]

[1] Department of Veterinary Medicine and Animal Productions, University of Naples "Federico II", 80137 Naples, Italy; angela.salzano@unina.it (A.S.); sara.damiano@unina.it (S.D.); livia.dangelo@unina.it (L.D.); neglia@unina.it (G.N.); roberto.ciarcia@unina.it (R.C.)
[2] Council for Agricultural Research and Economics (CREA)—Research Centre for Olive, Fruit and Citrus Crops, 95024 Acireale, Italy; gabriele.ballistreri@crea.gov.it
[3] Council for Agricultural Research and Economics—Research Centre for Animal Production and Aquaculture, 85051 Bella Muro, Italy; salvatore.claps@crea.gov.it (S.C.); drufrano@tiscali.it (D.R.)
[4] Department of Veterinary Medicine, University "Aldo Moro" of Bari, 70010 Valenzano, Italy; pasquale.depalo@uniba.it
* Correspondence: aristide.maggiolino@uniba.it
† The authors contributed equally to the work.

Citation: Salzano, A.; Damiano, S.; D'Angelo, L.; Ballistreri, G.; Claps, S.; Rufrano, D.; Maggiolino, A.; Neglia, G.; De Palo, P.; Ciarcia, R. Productive Performance and Meat Characteristics of Kids Fed a Red Orange and Lemon Extract. *Animals* **2021**, *11*, 809. https://doi.org/10.3390/ani11030809

Academic Editor: Gema Romero Moraleda, Carlos Sandoval Castro and Alberto Stanislao Atzori

Received: 8 February 2021
Accepted: 9 March 2021
Published: 13 March 2021

Publisher's Note: MDPI stays neutral with regard to jurisdictional claims in published maps and institutional affiliations.

Copyright: © 2021 by the authors. Submitted for possible open access publication under the terms and conditions of the Creative Commons Attribution (CC BY) license (http://creativecommons.org/licenses/by/4.0/).

Table S1. Effect of including RLE in the diet on kids' carcass measurements and cuts incidence. Group RLE (RLE treated group); Group CON (control).

	Group RLE	Group CON	SEM [1]	*p* value
Carcass measurements (cm)				
Carcass length	34.25	34.67	0.85	0.74
Internal carcass length	43.50	43.42	1.03	0.96
Leg length	26.50	26.42	0.61	0.92
Chest circumference	12.25	11.58	0.61	0.46
Chest width	13.83	12.92	0.49	0.20
Compactness indices (kg/m)				
Leg compactness	1.71	1.65	0.14	0.22
Carcass compactness	13.12	12.95	1.87	0.40
Cuts incidence (%)				
Hint limb	24.10	24.17	1.38	0.46
Front limb	24.86	24.62	1.29	0.35
Ribs	22.11	21.45	1.35	0.23
loin	14.23	14.58	0.98	0.40
Hip	2.86	3.18	0.27	0.74
Neck	9.71	10.52	0.69	0.84

[1] Standard error of the means.

Table S2. Effect of including RLE in the diet on kids' meat chemical composition (expressed as g/100g of meat). Group RLE (RLE treated group); Group CON (control).

	Group RLE	Group CON	SEM [1]	*p* value
Item (g/100g)				
Moisture	73.48	73.86	6.31	0.66
Proteins	22.10	21.83	3.05	0.61
Fat	1.52	1.54	0.14	0.42
Ash	1.20	1.36	0.19	0.38

	Group RLE	Group CON	SEM[1]	*p* value
Item (g/100g)				
Moisture	73.48	73.86	6.31	0.66
Proteins	22.10	21.83	3.05	0.61
Fat	1.52	1.54	0.14	0.42
Ash	1.20	1.36	0.19	0.38

[1] Standard error of the means.
